# Effects of Caffeine, Zinc, and Their Combined Treatments on the Growth, Yield, Mineral Elements, and Polyphenols of *Solanum lycopersicum* L.

**DOI:** 10.3390/antiox13091100

**Published:** 2024-09-11

**Authors:** Elena Vichi, Alessandra Francini, Andrea Raffaelli, Luca Sebastiani

**Affiliations:** 1 Institute of Crop Science (ICS), Scuola Superiore Sant’Anna, Piazza Martiri della Libertà 33, 56127 Pisa, Italy; elena.vichi@santannapisa.it (E.V.); andrea1.raffaelli@santannapisa.it (A.R.); luca.sebastiani@santannapisa.it (L.S.); 2 Institute of Agricultural Biology and Biotechnology—National Research Council (IBBA-CNR), Via Moruzzi 1, 56124 Pisa, Italy

**Keywords:** LC-MS/MS, biostimulant, flavonoids, target hazard quotients, translocation factor

## Abstract

(1) Background: The effects of Zn and caffeine as promoters of fruit quality in the *Solanum lycopersicum* L. cultivar ‘Panarea’ were tested. (2) Methods: During the 56 days of the experiment, plants were treated weekly with 100 mL of 1 mM Zn (Zn), 1 mg L^−1^ caffeine trimethyl-^13^C (caffeine), and 1 mM Zn + 1 mg L^−1^ caffeine trimethyl-^13^C (Zn + caffeine) and compared to plants that were given tap water (control). (3) Results: Caffeine was taken up by the roots and translocated to the leaves, which positively influenced the number of fruits per plant. After 56 days of treatment, Zn induced a positive increase in tomato dry weight, reducing shoot length (−16.7%) compared to the other treatments. Zn + caffeine had a positive effect on the phenylpropanoid pathway of fruits, and 4-coumaric acid, caffeic acid, and t-ferulic acid were significantly increased, as well as the total antioxidant capacity of the tomatoes. In the flavonoid pathway, only apigenin and luteolin contents were reduced by treatments. The tomatoes showed similar concentrations of the mineral elements Cu, Mn, Fe, Na, Ca, Mg, and K. The Zn and caffeine target hazard quotients were <1, indicating that health risks via the consumption of these tomatoes did not occur. (4) Conclusions: Tomato plants could be irrigated with water containing lower values of Zn, caffeine, and a combination of the two. The treated fruits are rich in antioxidant compounds, such as coumaric acid, caffeic acid, and t-ferulic acid, which are beneficial for human health. No considerable health risks associated with human consumption have been detected.

## 1. Introduction

The Food and Agriculture Organization (FAO) of the United Nations has emphasized the need for sustainable agricultural practices that can produce more food with fewer environmental impacts. Several key strategies can be implemented to achieve this goal, and the use of mineral elements and organic compounds represents an additional and increasingly important tool.

Among mineral elements, Zn is an essential micronutrient that plays a crucial role in various plant physiological processes. Zn is a co-factor in many important antioxidant enzymes in plants, such as copper/zinc-superoxide dismutase [1], and participates in several processes, such as germination and reproductive growth, with general effects on plant physiology and morphology [2]. The optimal level of Zn in crops ranges from 30 to 200 mg Zn kg^−1^ dry weight [3]. Zn deficiency is a well-known nutritional disorder that modifies human health, mainly around the world, where principal food crops are the key source of daily calorie intake [3]. For these reasons, the Zn fertilization of crops is an objective of research [4].

Caffeine (1,3,7-trimethylxanthine) is a natural alkaloid found in various plants, including coffee (*Coffea* spp.), tea (*Camellia sinensis*), cocoa (*Theobroma cacao*), and other species. Caffeine is also an ingredient in foods and pharmaceuticals recognized for its stimulant effects on humans and the most frequently detected compound in water, and for this, it is considered a trace of anthropogenic presence [5,6]. Water data indicate that caffeine is relatively stable, with a half-life ranging from 100–240 days to 10 years [7,8]. 

In plants, it has been observed that caffeine effects are related to dose and species, showing clear biostimulant effects at low doses. Pierattini et al. [9] demonstrated that the treatment of *Populus alba* cv ‘Villafranca’ with 2 mg L^−1^ caffeine (trimethyl-^13^C) leads to a significant increase in stem and leaf growth. Caffeine can be used as a biostimulant in cucumbers, spinach, and lentils [10,11,12]. In *Phaseolus vulgaris*, caffeine increased the mitosis of the root meristematic cells [13], whereas, in *Capsicum annum*, low doses of caffeine (between 2 and 38 µM) increased the mean height of these plants [14]. In spinach (*Spinacia oleracea* L.), caffeine significantly reduced Cd stress, increasing the accumulation of osmolytes and antioxidant molecules, such as proline and ascorbic acid, and reducing lipid peroxidation and H_2_O_2_ content [11]. 

The application of low concentrations of caffeine and Zn has been a subject of interest for enhancing plant growth and development [12,15,16,17]. Combining these two substances can offer synergistic effects, potentially leading to improved plant health and productivity. This application appears interesting in tomato (*Solanum lycopersicum* L.), which is one of the most popular and widespread horticultural crops worldwide. Moreover, tomato plants represent an ideal research model because of their roots, stems, leaves, and edible fruit organs.

According to the Food and Agriculture Organization (FAO), it is cultivated in an area of approximately 5 million hectares, with an annual production of 186 million tons [18]. Considering the increased daily consumption and intrinsic qualities of tomatoes, they play a role in human health. Tomato is a well-characterized source of carotenoids and polyphenols, which are considered targets for cancer prevention [19]. According to their core structures, these antioxidant molecules can be divided into different groups, such as anthocyanins, hydroxycinnamic acids, flavonols, and flavanones. In addition, two flavonol glycosides, rutin and kaempferol-3-rutinoside, are present in tomatoes [19].

It is important to consider that high levels of both Zn and caffeine can be toxic to plants. When Zn reaches toxic concentrations, its effects range from a decrease in biomass to chlorosis, followed by necrosis, to hypoxic-like responses [20,21,22,23,24]. Similarly, a concentration of 2.5 M caffeine inhibits root mitosis in rice [25], and retardation in the seedling growth of *Arabidopsis* and tobacco has been observed when grown under 1 mM caffeine [26]. High levels of Zn and caffeine uptake by plants can also pose a risk for the tomato consumer, exceeding the safety limit for humans [27,28], and the non-carcinogenic target hazard quotient (THQ) [29] can be used. 

In this study, we aimed to explore the potential roles of Zn and caffeine in improving tomato plant yield, mineral nutrition, antioxidant capacity, and polyphenol profile. We hypothesized that Zn and caffeine would exert a positive effect on tomato plants. We tested the following: (1) the positive effects of Zn and caffeine on tomato yield, phytochemical parameters such as polyphenols, antioxidant capacity, ethylene production, and solid soluble content (Brix); (2) the mineral nutrient profile of whole plants and fruits; and (3) the risk for humans consuming tomato fruits that could accumulate Zn and caffeine. 

## 2. Materials and Methods

### 2.1. Plant Materials and Treatments

Red tomatoes (*Solanum lycopersicum* L.) cv ‘Panarea’ were transplanted (one plant for pot) in 7 L pots filled with a peat-based commercial substrate (Potgrond H 90 Klasmann-Deilmann Belgium N.V., Bolzano, Italy). Each plant was fertilized at the start of the experiment with 2.5 g of NPK-MgO (16/10/18/2–Cifo, Orto, Bologna–Italy) and grown in a greenhouse (N.43.76926550560972, W.10.407108271387008, San Giuliano Terme, Italy) from April to July. After three weeks, the plants were divided into groups (*n* = 7) and treated weekly until the end of the trial (56 days) with 100 mL of tap water (control, containing 65 ± 13 µg L^−1^ of Zn), 1 mM Zn (0.136 mg L^−1^) (Zn), 1 mg L^−1^ caffeine trimethyl-^13^C (caffeine), and 1 mM Zn + 1 mg L^−1^ caffeine trimethyl-^13^C (Zn + caffeine). Regular daily irrigation with tap water was performed according to the plant growth dynamics. Zn was distributed in the form of ZnCl_2_ (anhydrous powder, ≥98%, Sigma-Aldrich, Milan, Italy). Caffeine trimethyl-^13^C (powder, ≥99, Sigma-Aldrich, Milan, Italy) was used to discriminate between exogenous and endogenous caffeine (Figure S1). 

### 2.2. Yield Measurements and Sampling

Stem length of each plant was measured weekly (*n* = 7). During the experiment, the first, second, and third trusses were collected at 32, 48, and 56 days from the beginning of the experiment. The total yield and fruit number for each plant were determined by weighing and counting all fruits. For each truss, the first and second fruits of the truss were collected and analyzed. For the first fruit, the fresh weight (FW), dry weight (DW), caliber (cm), total soluble solids (°Brix, using a portable refractometer Shodex, West Berlin, NJ, USA), mineral element, caffeine, and polyphenol concentrations were determined. For the second fruit, ethylene analyses were performed. After 56 days (last sampling), roots, stems, and leaves were collected, and FW was measured and stored for specific analysis. Part of the plant material was oven-dried at 60 °C until its weight remained constant.

### 2.3. Ethylene Measurements

Ethylene production in the first, second, and third trusses was assessed by enclosing the second fruits of each truss in airtight glass bottles sealed with plastic screwcaps and fitted with rubber septa. Incubation lasted for 1 h, and the headspace (1 mL) was removed from the jar and injected into a gas chromatograph (HP5890, Hewlett-Packard, Menlo Park, CA, USA) equipped with a flame ionization detector and metal column (internal dimension 150 × 0.4 cm) packed with Hysep T. Column, and detector temperatures were set at 70 and 350 °C, respectively. The nitrogen carrier gas was used at a flow rate of 30 mL min^−1^. The ethylene data (*n* = 5) are expressed as nl g^−1^ h^−1^.

### 2.4. Zinc and Mineral Element Analyses

A microwave-assisted digestion method was used to digest 0.3 g of the dried first fruit of the first, second, and third trusses and leaf, stem, and root samples with 8 mL of 65% nitric acid (Sigma-Aldrich, Milan, Italy). The mixture was digested using a COOLPEX Smart Microwave Reaction System (Yiyao Instrument Technology Development Co. Ltd., Shanghai, China). The digested solution was diluted to 30 mL with deionized water. Mineral element quantification was performed using a Microwave Plasma-Atomic Emission Spectrometer (4210 MP-AES, Agilent Technologies, Santa Clara, CA, USA). The wavelengths used were 213.8 nm for Zn, 393.3 nm for Ca, 766.4 nm for K, 588.9 nm for Na, 324.8 nm for Cu, 371.9 nm for Fe, 403.0 nm for Mn, and 285.2 nm for Mg. *Daucus carota* (L.) leaf tissue was used as the analytical standard reference (WEPAL IPE, Wageningen University, Wageningen, Netherlands). A multi-element standard solution was prepared in 5% HNO_3_ (*v*/*v*) medium and diluted with Milli-Q H_2_O for mineral quantification. The limits of detection (LOD) were calculated as three times the standard deviation of the blank samples: Zn 7.8, Ca 39, K 41.2, Na 35.5, Cu 0.9, Fe 1.6, Mg 0.8, and Mn 0.6 mg kg^−1^. The results (*n* = 7) are expressed on a dry mass basis (mg kg^−1^ DW). 

### 2.5. Caffeine-^13^C Extraction and Polyphenol Extraction

Fresh roots (0.5 g), stems, leaves, and first fruits of the first, second, and third trusses were extracted after grinding in mortars with 3 mL of 0.1% formic acid (Sigma-Aldrich, Milan, Italy) and 3 μL of 15% hydrochloric acid (Sigma-Aldrich, Milan, Italy). The extract was centrifuged at 17,000× *g* for 5 min (Allegra 64R, Beckman Coulter Inc., Brea, CA, USA), and the supernatant was filtered through 0.20-μm syringe filters (Sartorius Stedim Biotech GmbH, Gottingen, Germany) before storing at −20 °C until liquid chromatography–tandem mass spectrometry (LC-MS/MS) analysis. The fresh fruit in first position of first, second, and third truss clusters was used to determine the selected polyphenols: protocatechuic acid (PCTA), 4-coumaric acid (PCA), caffeic acid (CFA), t-ferulic acid (TFRA), naringenin (NRG), apigenin (APG), luteolin (LTO), quercetin (QCT), chlorogenic acid (CGA), piceid (PCD), phloridzin (PDZ), kaempferol 7-G (QCT7G), kaempferol 3-G (QCT3G), kaempferol 3-O-rutinoside (KPF3R), rutin (RTN), and quercetin 3,4 DG (QCTDG) (Sigma-Aldrich, Milan, Italy). Tomato samples (7.5 g FW) were extracted with 25 mL of 80% methanol (Sigma Aldrich, Milan, Italy) for 30 min at room temperature (25 °C) using a laboratory shaker (VDRL mod. 711/CS ASAL, Milan, Italy). Extracts were filtered through a 0.45 μm pore size membrane (Sigma-Aldrich, Milan, Italy) before injection into LC-MS/MS mass spectrometer (AB Sciex LLC, Framingham, MA, USA). Analytical standards for caffeine (trimethyl-^13^C) and specific polyphenols were used as the calibration curves. The matrix effects and recovery efficiencies were also evaluated.

### 2.6. Caffeine and Selected Polyphenols Analyses

Caffeine (trimethyl^13^-C) and selected polyphenol concentrations in the fruits were determined by LC-MS/MS mass spectrometry (Sciex 5500 QTrap+) using an information-dependent acquisition (IDA) method with selected reaction-monitoring (SRM) transitions per component as a survey scan and MS-MS enhanced product ion (EPI) spectrum acquisition. An AB Sciex 5500 QTrap+ mass spectrometer (AB Sciex LLC, Framingham, MA, USA), equipped with a Turbo V ion spray source coupled to an ExionLC AC System custom-made by Shimadzu (Shimadzu Corporation, Kyoto, Japan), was used to determine the specific molecules.

### 2.7. HPLC-MS/MS Method for Polyphenols

A Phenomenex Kinetex ^®^ Biphenyl 100 × 2.1 mm, 2.6 µm particle size column (Phenomenex, Torrance, CA, USA) was employed for the chromatographic separation. An elution gradient was performed using acetonitrile containing 0.1% *v*/*v* formic acid and Milli-Q water with 0.1% *v*/*v* formic acid (Sigma-Aldrich, Milan, Italy). MS-MS detection was performed in negative ion mode. The common source parameters were as follows: nebulization gas (GS1) 50, turbo gas (GS2) 50, curtain gas (CUR) 10, temperature (TEM) 500 °C, ion spray voltage (IS) −4500 V, and input potential (EP) 10 V. The declustering potential (DP), collision energy (CE), and collision cell exit potential (CXP) were adjusted for the specific SRM for each component. The SRM transitions and corresponding compound parameters are listed in Table S1.

### 2.8. HPLC-MS/MS for Caffeine

An Agilent PhenylHexyl 2 × 100 mm 2.7 µm particle size column (Agilent, Santa Clara, CA, USA) was employed for chromatographic separation. An elution gradient was performed using acetonitrile containing 0.1% *v*/*v* formic acid and Milli-Q water (Merck KGaA, Darmstadt, Germany) with 0.1% *v*/*v* formic acid (Sigma-Aldrich, Milan, Italy). Tandem mass spectrometry (MS-MS) was performed in the positive ion mode. The common source parameters were as follows: nebulization gas (GS1) 50, turbo gas (GS2) 45, curtain gas 25, temperature 500 °C, ion spray voltage 5500 V, and input potential 10 V. The compound parameters were adjusted for the selected reaction-monitoring transitions for each component and are shown in Table S1.

### 2.9. DPPH Assay

The scavenging activity of 1,1-diphenyl-2-picrylhydrazyl (DPPH) radical tomatoes was analyzed with 200 μL of the methanol extract previously used for polyphenol assay, mixed with 800 μL of a Tris-HCl 100 mM solution (Sigma-Aldrich, Milan, Italy), pH 7.0, and finally kept in the dark for 30 min after adding 250 µM DPPH (Sigma-Aldrich, Milan, Italy). Methanol: water (80:20, *v*:*v*) was used as a control reference, and the absorbance was measured at 517 nm using a spectrophotometer (Infinite 200 PRO, Tecan Italia Srl Milan, Italy). The radical scavenging activity of the extracts was calculated using the following equation:

$$DPPH\;inhibition\left(\%\right)=\frac{Absorbance\;of\;control-Absorbance\;of\;sample}{\begin{array}{c} \mathrm{Absorbance}\;\mathrm{of}\;\mathrm{control} \end{array}}\times 100$$

### 2.10. Translocation Factor 

The response of tomato plants (*n* = 7) to Zn and Zn + caffeine application was evaluated in terms of the translocation factor (Tf), a unit-less index indicating the ability of the plant to transfer caffeine or Zn from roots to aerial part of tomato plants. Tf was calculated to evaluate the capability of plants to accumulate Zn or caffeine, absorbed by roots, in the aerial parts (stems, leaves, and fruits harvested after 56 days of treatment). Tf was calculated using the following equation:

$$Tf=\frac{Zn\;or\;Caffeine\;concentration\;in\;the\;aerial\;parts\;(mg\;{kg}^{-1})}{Zn\;or\;Caffeine\;concentration\;in\;the\;root\;parts\;(mg\;{kg}^{-1})}$$

### 2.11. Risk Assessment

The health risks caused by the intake of tomato fruits grown under Zn and caffeine treatments were assessed using the THQ index [29]. THQ was calculated as the ratio of exposure to Zn or caffeine to the reference dose (RfD), which is the highest level at which no adverse health effects are expected. THQ describes the non-carcinogenic health risks posed by exposure to Zn or caffeine. If THQ is <1, non-carcinogenic health effects are expected. In contrast, a THQ >1 indicates that there is a possibility that adverse health effects could occur. THQ was calculated as follows:

$$THQ=\frac{Efr\times Ed\times Fir\times C}{RfD\times Bw\times ATn}\times 10^{-3}$$

where Efr = exposure frequency (365 days/year); Ed = exposure duration (50 years); RfD = reference dose (being 0.3 mg kg^−1^ day^−1^ for Zn and 37 mg kg^−1^ day^−1^ for caffeine according to EFSA and Antoine et al. [28,29]. Bw = the estimated average body weight (70 kg) considering exposed consumers aged 15–17 years old; ATn = average time of exposure to non-carcinogenic HMs (Ed × 365 days/year), C = concentration of Zn or caffeine in tomato fruit; Fir = food ingestion rate in grams per day (for 50 g of tomato per day).

### 2.12. Statistical Analysis

Before performing the statistical test, the normality of the data was assessed and analyzed using two-way ANOVA. Tukey’s post-hoc test for post-hoc mean comparison at *p* = 0.05 was used. *t*-test analyses were performed to determine the differences between the control and treated plants. A heat map and principal component analysis (PCA) were performed for the mineral elements and polyphenols. Graphs and statistical analyses were performed using Prism-GraphPad 10.1 Mac.

## 3. Results

**Figure 1:**
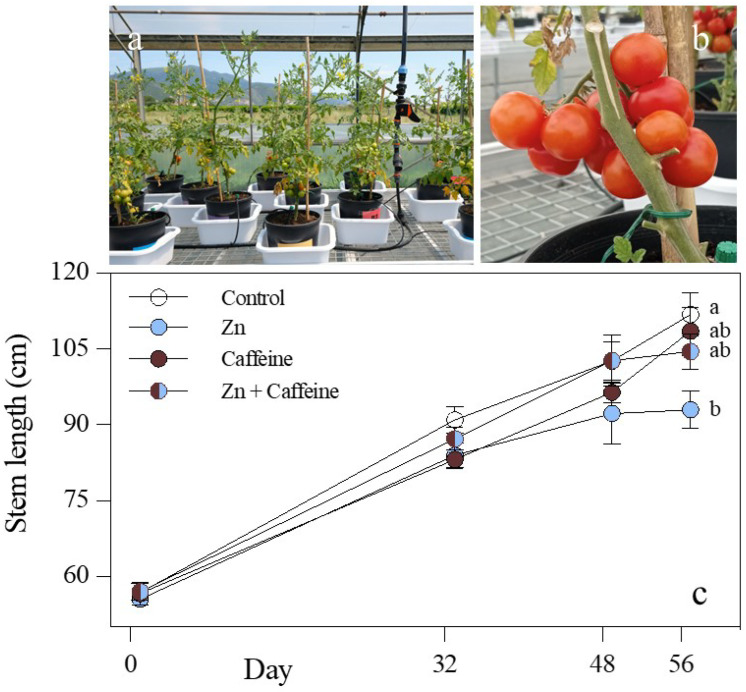
(**a**) Overview of plants (*Solanum lycopersicum* cv ‘Panarea’) grown under greenhouse conditions; (**b**) tomato truss; and (**c**) stem length at 0, 32, 48, and 56 days of the experiment. Plants were treated with tap water (control), 0.136 mg L^−1^ Zn (Zn), 1 mg L^−1^ caffeine-(trimethyl-^13^C) (caffeine), and 1 mg L^−1^ caffeine (trimethyl-^13^C) + 0.136 mg L^−1^ Zn (Zn + caffeine). Data represent the mean ± SD (*n* = 7). The data followed a normal distribution and were subjected to two-way ANOVA, and the values indicated with different letters were significantly different from each other following Tukey’s post-hoc test, *p* ≤ 0.05 (Supplementary Table S2).

One of the main objectives of this study was to assess whether Zn and caffeine have a positive effect on tomato plant performance, with a focus on fruit quality and safety. The macroscopic observation of tomato plants demonstrated that caffeine application did not change the growth performance compared with the control (Figure 1), either added to Zn or alone (Figure 1). In contrast, plant height was significantly lower in Zn-treated plants than in the control plants, and stem length was significantly reduced by 16.7% (Figure 1c and Table S2).

**Figure 2:**
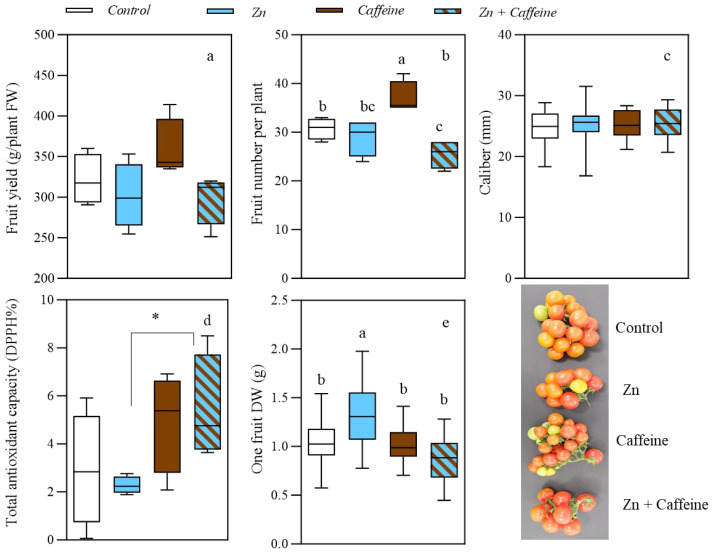
Box plot representation of the effects of treatments with tap water (control), 0.136 mg L^−1^ Zn (Zn), 1 mg L^−1^ caffeine-(trimethyl-^13^C) (caffeine), and 1 mg L^−1^ caffeine (trimethyl-^13^C) + 0.136 mg L^−1^ Zn (Zn + caffeine) on the fruit of *Solanum lycopersicum* cv ‘Panarea’ after 56 days of the experiment. (**a**) Fruit yield (*n* = 7); (**b**) fruit number per plant (*n* = 7); (**c**) caliber (*n* = 7); (**d**) total antioxidant capacity (DPPH%) (*n* = 4); (**e**) one fruit dry weight of the first tomato per truss (*n* = 7). The data followed a normal distribution and were subjected to two-way ANOVA (Supplementary Table S3). Values indicated with different letters differ significantly from each other (Tukey’s post-hoc test, *p* ≤ 0.05). *t*-test analyses * = *p* <0.05.

Tomato fruits were assessed at harvest for (i) fruit yield, (ii) fruit number per plant, (iii) caliber, (iv) total antioxidant capacity (DPPH%), and (v) fruit dry weight of the first tomato per truss. For the total fruit yield per plant, there was a not significant increase of 6% or 37 g/plant due to caffeine treatment (Figure 1a) compared to the control; these data are promising and deserve future experimental work. The number of fruits per plant was the highest in caffeine-treated plants (Figure 2b), with a significant interaction between Zn and caffeine factors (*p* = 0.009, Table S3). The fruit number per plant increased under caffeine treatments by 20% compared to the control, whereas a slight reduction was observed under Zn + caffeine treatments (−17%). There were no significant differences in caliber between the treatment and control groups (Figure 2c). Regarding the total antioxidant activity of tomato fruits after 56 days of treatment (Figure 2d, Table S3), an increase was detected considering the caffeine factor (*p* = 0.019). The results indicated that tomato plants treated with caffeine increased their average antioxidant capacity by 69% and 85% compared to the average control in the caffeine and Zn + caffeine treatments, respectively (Figure 2d). Finally, Zn application significantly increased the fruit DW when compared with the other treatments (+21%, +23%, and +34% relative to the control, caffeine, and Zn + caffeine, respectively) (Figure 2e, Table S3).

The total soluble solid content during the three harvesting periods ranged from 8.4 to 10.4 °Brix (Table 1), while concerning ethylene, the average values of the three harvesting periods ranged from 0.14 to 0.52 nl g^−1^ h^−1^ (Table 1 and Table S4). The total soluble solid content and ethylene concentration were not significantly affected by Zn or caffeine treatments (Table 1). 

**Figure 3:**
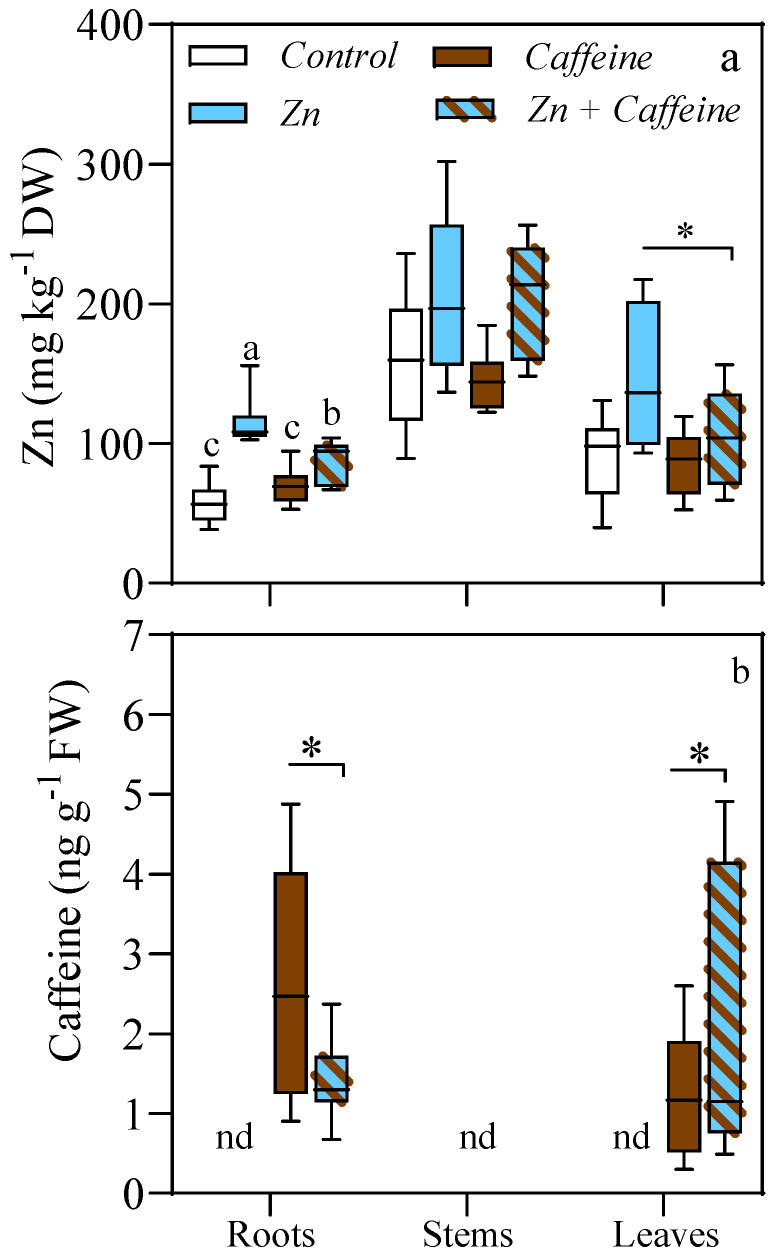
(**a**) Zn concentration (mg kg^−1^ DW) and (**b**) caffeine concentration (ng g^−1^ FW) in the roots, stems, and leaves of *Solanum lycopersicum* cv ‘Panarea’ after 56 days of treatment with tap water (control), 0.136 mg L^−1^ Zn (Zn), 1 mg L^−1^ caffeine-(trimethyl-^13^C) (caffeine), and 1 mg L^−1^ caffeine-(trimethyl-^13^C) + 0.136 mg L^−1^ Zn (Zn + caffeine). Statistical significances were determined with two-way ANOVA (data n = 7), and different letters indicate a statistical difference according to Tukey’s multiple comparison test (*p* ≤ 0.05) (Supplementary Table S5); nd = not detected; * = *p* < 0.05.

The uptake of Zn and caffeine in each organ of the tomato plants was evaluated (Figure 3). The stem is the organ in which Zn reached the highest value compared to roots and leaves (204 and 200 mg kg^−1^ DW in plants treated with Zn + caffeine and Zn alone) (Figure 3a and Table S5). ANOVA did not show any significant interaction between Zn and caffeine in the stems (*p* = 0.838). In the roots, ANOVA revealed a significant interaction between Zn and caffeine (*p* = 0.002) (Table S5). A significant increase of 53% and 98% in root Zn concentration was observed in plants treated with Zn + caffeine (89 ± 14.9 mg kg^−1^ DW) and Zn alone (115 ± 17.3 mg kg^−1^ DW) in comparison to the control plants (Figure 3a). The *t*-test analyses between Zn and Zn + caffeine treatments indicated that in roots (*p* = 0.041) and leaves (*p* = 0.035), the treatment with Zn + caffeine reduced the concentration of Zn allocated to these two organs (Figure 3a). When present in treatments, caffeine was taken up by the roots (2.6 ± 1.51 and 1.4 ± 0.53 ng g^−1^ FW under caffeine and Zn + caffeine treatments respectively) and translocated to the leaves (1.3 ± 0.28 and 3.5 ± 1.28 ng g^−1^ FW under caffeine and Zn + caffeine treatments, respectively) (Figure 3b). Under our experimental conditions, caffeine was not detected in the stems (Figure 3b). In particular, the *t*-test indicated that at the root level, the caffeine concentration was 4.5 times lower (*p* = 0.031) under the Zn + caffeine treatments than under caffeine alone. In contrast, mixed Zn + caffeine exposure increased caffeine translocation to the leaves (*p* = 0.037, Figure 3b). 

**Figure 4:**
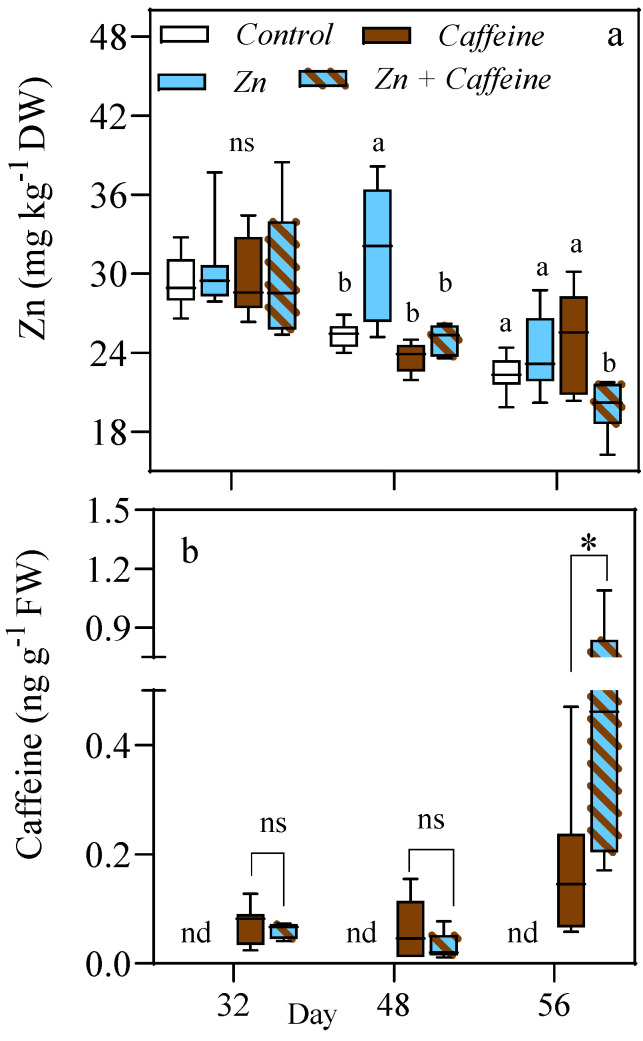
(**a**) Zn concentration (mg kg^−1^ DW); (**b**) Caffeine concentration (ng g FW^−1^) in tomato fruits *Solanum lycopersicum* cv ‘Panarea’ after 32, 48, and 56 days of treatments with tap water (control), 0.136 mg L^−1^ Zn (Zn), 1 mg L^−1^ caffeine-(trimethyl-^13^C) (caffeine), and 1 mg L^−1^ caffeine-(trimethyl-^13^C) + 0.136 mg L^−1^ Zn (Zn + caffeine). Statistical significances were determined with two-way ANOVA (data *n* = 7), and different letters indicated a statistical difference according to Tukey’s multiple comparison test (*p* ≤ 0.05) (Supplementary Table S6); ns = not significant; nd = not detected; * = *p* < 0.05. *t*-test between caffeine and mix results was also performed.

After verifying the uptake of Zn and caffeine in the non-edible parts of the plant, we focused on the fruits during the three different harvesting times after 32, 48, and 56 days of treatment (Figure 4a). The average Zn concentration in tomatoes ranges from a minimum of 19.7 to a maximum of 31.6 mg kg^−1^ DW. A higher average value was detected in Zn + caffeine fruits after 56 days of treatment, whereas it was lower in fruits after 48 days of Zn treatment (Figure 4a). No significant changes (*p* = 0.988) in the Zn concentration in fruits were observed between the treatments at the first (32 days) harvest time (Table S6). In contrast, ANOVA showed a significant difference (*p* = 0.027) in the uptake of Zn between treatments at the second harvest time (48 d), with a significantly higher uptake of Zn in plants exposed to 0.136 mg L^−1^ of Zn. Finally, in plants treated with Zn + caffeine, after 56 days of exposure, we observed a significant reduction (*p* = 0.003) of 12% in the Zn content of the fruits (Figure 4a). 

The uptake and accumulation of caffeine in the vegetables indicated that tomatoes could take up and accumulate this molecule in the soil. The amount of caffeine increases with exposure time; in fact, the average values for caffeine after 32 and 48 days range between 0.06 and 0.07 (ng g^−1^ FW), while a maximum value of 0.52 ng g^−1^ FW was measured after 56 days of Zn + caffeine exposure (Figure 4b).

**Figure 5:**
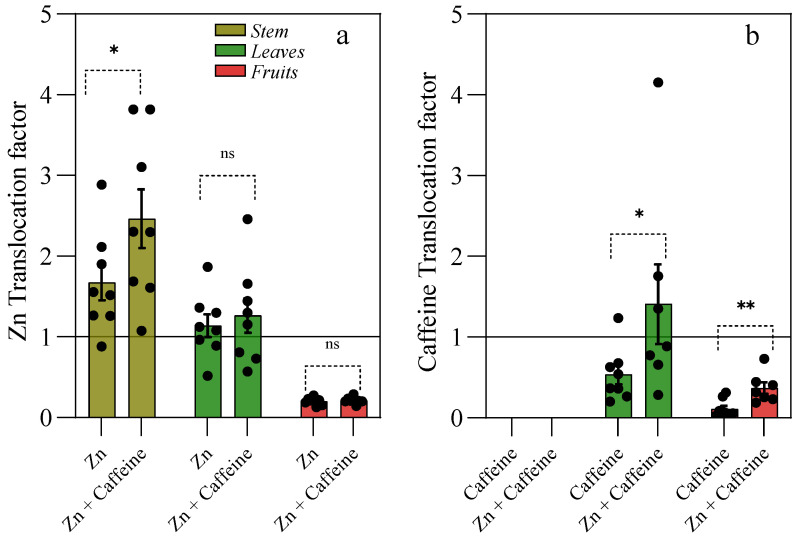
Translocation factors for Zn (**a**) and caffeine (**b**) in *Solanum lycopersicum* ‘Panarea’ stem, leaves, and tomatoes after 56 days of treatment with 0.136 mg L^−1^ Zn (Zn), 1 mg L^−1^ caffeine-(trimethyl-^13^C) (caffeine), 1 mg L^−1^ caffeine (trimethyl-^13^C), and 0.136 mg L^−1^ Zn (Zn + caffeine). Data were analyzed by *t*-test, and significant results are reported (* = *p* < 0.05; ** = *p* < 0.01; ns = not significant).

Under our experimental conditions, the ratio of Zn that reached the aerial part with respect to that present in the roots (Tf) varied among different organs, with values higher than 1 in the stems and leaves (Figure 5a). The highest Zn Tf values were observed in the stems of the plants treated with Zn + caffeine (Tf = 2.4; *p* = 0.041). No significant Tf was found in the leaves or fruits (Figure 5a). The calculation of caffeine Tf plants showed Tf <1 in the leaves and fruits, with significant values in both the leaves and fruits under Zn + caffeine treatment (Figure 5b).

Data and statistical analyses of the concentrations of mineral elements in the first-, second-, and third-truss fruits are shown in Tables S7 and S8. 

**Figure 6:**
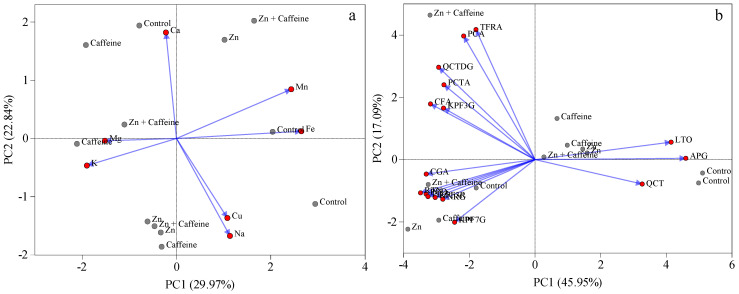
Principal component analysis (PCA) biplot of (**a**) mineral elements analyzed in the first fruit of the first, second, and third trusses (Supplementary Tables S7 and S8). Principal component analysis (**b**) of selected polyphenol–protocatechuic acid (PCTA), 4-coumaric acid (PCA), caffeic acid (CFA), trans-ferulic acid (TFRA), naringenin (NRG), apigenin (APG), luteolin (LTO), quercetin (QCT), chlorogenic acid (CGA), piceid (PCD), phloridzin (PDZ), kaempferol 7-G (QCT7G), kaempferol 3-G (QCT3G), kaempferol 3-O-rutinoside (KPF3R), rutin (RTN), quercetin 3,4 DG (QCTDG) of *Solanum lycopersicum* cv ‘Panarea’ treated with tap water (control), 0.136 mg L^−1^ Zn (Zn), 1 mg L^−1^ caffeine (trimethyl-^13^C) (caffeine), 1 mg L^−1^ caffeine (trimethyl-^13^C), and 0.136 mg L^−1^ Zn (Zn + caffeine). The loadings (red color) and score (grey color) of the PCA are reported.

All tomatoes analyzed had similar concentrations of the mineral elements Cu, Mn, Fe, Na, Ca, Mg, and K after 32, 48, and 56 days of treatment, as indicated by the biplot data of the PCA analyses (Figure 6a). The scores of each tomato sample were examined in a two-dimensional plot of the first two principal components (52.8% of the total variability), and no separation of the samples into groups was found. K was the most abundant element in third-truss fruits, with concentrations ranging from 27,159 to 32,951 mg kg^−1^ DW (Figure 6a). In the first and second truss, K ranged from 27,585 to 32,362 mg kg^−1^ DW and 27,691 to 31,887 mg kg^−1^ DW, respectively (Table S7). The Mg and Ca values did not differ significantly (*p* > 0.05) between the treatments. 

The ANOVA analysis of mineral element data in fruits after 32, 48, and 56 days, in general, did not reveal significant differences in the interactions between the Zn and caffeine factors at the first and third truss harvests (Table S8). The only exceptions were Mn (Zn × caffeine, *p* = 0.027), Fe (Zn × caffeine, *p* = 0.047), and Na (Zn × caffeine *p* = 0.049) after 48 days of treatment. Some other interesting statistical differences were observed at the 56th day of treatment for Fe (Zn factor, *p* = 0.006) (Table S8).

**Figure 7:**
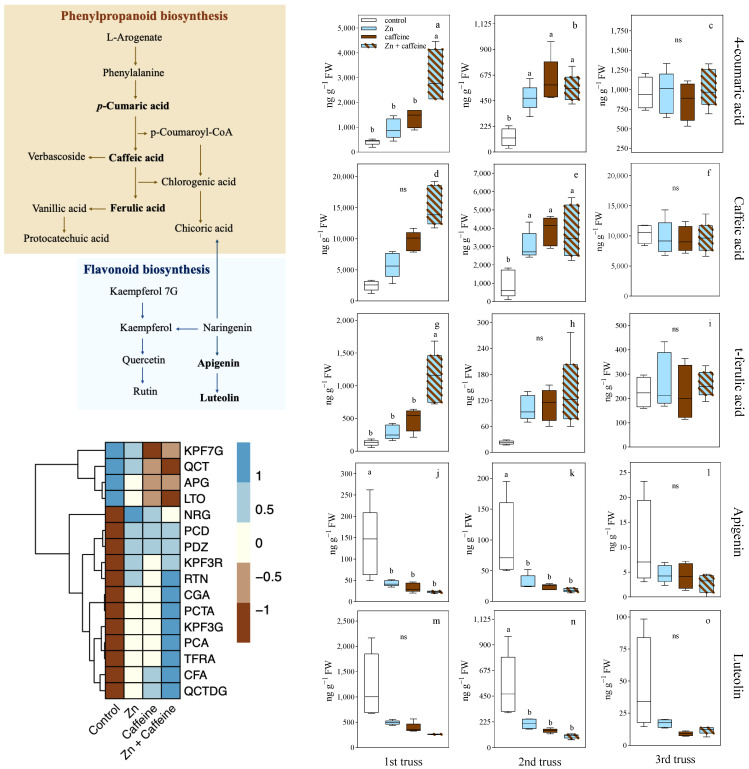
Schematic representation of the putative biosynthetic pathways of the main secondary compounds and hierarchical clustering analysis (HCA) plot of polyphenols in *Solanum lycopersicum* cv ‘Panarea’; box plot representation of 4-coumaric acid (PCA) (**a**–**c**), caffeic acid (CFA) (**d**–**f**), t-ferulic acid (TFRA) (**g**–**i**), apigenin (APG) (**j**–**l**), and luteolin (LTO) (**m**–**o**) in *Solanum lycopersicum* cv ‘Panarea’ after 32, 48, and 56 days of treatment with tap water (control), 0.136 mg L^−1^ Zn (Zn), 1 mg L^−1^ caffeine-(trimethyl-^13^C) (caffeine), and 1 mg L^−1^ caffeine-(trimethyl-^13^C) + 0.136 mg L^−1^ Zn (Zn + caffeine). Statistical significances were determined with two-way ANOVA (*n* = 5) and different letters indicated a statistical difference according to Tukey’s multiple comparison test (*p* ≤ 0.05) (Supplementary Table S9); ns = not significant.

In tomatoes, antioxidant molecules play a key role in determining fruit quality. The concentrations of the 16 polyphenols studied by UHPLC-ESI-MS/MS in all analyzed trusses are reported in Table 2 and Figure 6b and Figure 7. The content of 11 antioxidant molecules did not change in relation to the treatments applied (Table 2 and Table S9). Rutin was the most abundant polyphenol in the fruit, with an average of 207,640 ± 131,711 ng g^−1^ FW, followed by chlorogenic acid (91,221 ± 59,534 ng g^−1^ FW), kaempferol 3-R (8388 ± 9428 ng g^−1^ FW), and caffeic acid (6904 ± 4563 ng g^−1^ FW) (Table 2). 

The five significantly different UHPLC-ESI-MS/MS data corresponding to 4-coumaric acid, caffeic acid, t-ferulic acid, apigenin, and luteolin analyzed in the first fruits of the first, second, and third trusses were incorporated in the plant metabolic pathway overview to provide evidence for changes in the antioxidant production of the polyphenols studied. The biosynthetic routes are indicated by lines (Figure 7). Globally, in the phenylpropanoid pathway, 4-coumaric acid, caffeic acid, and t-ferulic acid were significantly increased by Zn or caffeine (Figure 7a–i, Table S9), whereas the flavonoid pathway (apigenin and luteolin) was significantly reduced by treatment (Figure 7j–o, Table S9). Treatment with Zn + caffeine increased the concentrations of 4-coumaric acid, caffeic acid, and t-ferulic acid compared with the control tomatoes at the first and second times of tomato harvest (Figure 7 and Table S9). Apigenin and luteolin levels decreased after Zn and caffeine treatments (Figure 7 and Table S9).

Principal component analysis was applied to the tomato metabolite dataset under different treatments (Figure 6b). When the scores of each tomato sample were examined in a two-dimensional plot of the first two principal components (63.04% of the total variability), a clear separation of samples into groups was found, with a particular correlation among protocatechuic acid, 4-coumaric acid, caffeic acid, trans-ferulic acid, and quercetin 3,4 DG with Zn + caffeine treatment (Figure 6b). A positive correlation was detected between luteolin and apigenin and control tomatoes; these are, in fact, the two polyphenols that were significantly reduced under the treatments (Figure 6b).

In Table 3, the average Zn and caffeine concentrations and THQ values for the first, second, and third truss clusters are shown. Remarkably, no specific THQ related to Zn or caffeine was >1. For Zn, the THQ values ranged from 0.006 to 0.009, and 0.003 and 0.005 under Zn and Zn + caffeine treatments, respectively. Concerning caffeine, the THQ values ranged from 1.44 × 10^−5^ to 4.33 × 10^−5^ for Zn and from 8.16 × 10^−6^ to 1.10 × 10^−4^ under Zn + caffeine treatment (Table 3).

## 4. Discussion

In horticulture, to use irrigation water supplemented with ions and molecules that could have a role as biostimulants, we need proof of the effective changes it can have on plant growth, fruit quality, and toxicity risks. The combined application of caffeine and zinc could stimulate overall metabolic processes, with the final goal of inducing plant growth and producing higher-quality fruits with improved size, mineral elements, and nutritional value.

It has been demonstrated that treatment with caffeine has a positive effect (biostimulator) on the number of fruits per plant, as observed by Jené et al. [30] on lentil yields, which showed an almost 50% increase when plants were treated with 10^−3^ M caffeine. These authors also observed that the effects of caffeine disappeared when an additional treatment was added to the plant, which could explain why the plants treated with the combination of Zn + caffeine did not show the same results as those treated with caffeine alone. Caffeine has a positive effect on the growth of *Vigna radiata* plants, as they grow faster in soil with caffeine [31]. Moreover, caffeine can inhibit seed germination but does not impair plant development when sprayed on plants or used to wet the soil [32]. Once caffeine treatment is applied, this compound in tomato is mainly absorbed in the roots; however, its translocation to the aerial part seems to be facilitated by a caffeine–Zn complex, as observed in our previous study on poplar plants [33]. When caffeine was present with Zn in the treatments, it also improved the total antioxidant capacity of fruits. Tomato plant organs have different capabilities to uptake Zn, as indicated by our results, but fruits have less ability to accumulate Zn because of the high presence of phloem tissue [34]. In this study, we showed that Zn was translocated to all organs in *S. lycopersicum* cv ‘Panarea’. The concurrent presence of Zn and caffeine reduces the translocation of this element at the fruit level. Zn can easily form complexes with several molecules, including organic acids such as citrate and malate, or amino acids such as histidine. Moreover, it has been reported that when a formulation containing caffeine and Zn is prepared, a Zn–caffeine complex is formed in the solution [35]. Under our experimental conditions, this complex could reduce Zn uptake in plants, as also reported in our previous observations in *Populus alba* leaves exposed to Zn + caffeine treatments [33]. The Zn concentration found in the stems under our experimental conditions did not explain the shoot reduction observed after 56 days. Zn is necessary for the synthesis of tryptophan, a precursor of indole acetic acid; therefore, it plays an active role in the production of auxin, an essential growth hormone [36] that may indirectly interfere with internodal elongation. 

Because tomato quality is also related to the content of different mineral elements that could contribute to taste, texture, and nutritional value [37] and play a role in antioxidant defense enzymes, the contents of Fe, Mn, Cu, and Ca were measured in this study. We hypothesized a positive effect of Zn and caffeine on tomatoes in terms of mineral elements; however, we found that treatments did not significantly interfere with mineral elements in tomatoes, and in general concentrations, the data are in line with the literature [38]. In the presence of Fe, the Zn–caffeine complex is converted into an Fe–caffeine complex [35], which could explain why Fe in fruit is reduced after 56 days of Zn + caffeine treatment. Regarding the total soluble content and ethylene production, our data were similar to those obtained by Roohanitaziani et al. [39], who measured the °Brix of 107 tomato accessions, and the Brix values range from 3.5–9.8. As ethylene promotes the ripening of tomato fruit, our data demonstrate that Zn + caffeine treatments did not interfere with the maturation process. The qualitative analysis of the phenolic compounds obtained in our study was consistent with those reported in the literature. For example, rutin and naringin have been reported as the main flavonoids in different varieties of red tomatoes [40,41,42].

It is interesting to note that the presence of Zn + caffeine in tomato plants induces an increase in the abundance of polyphenols like 4-coumaric, caffeic, and t-ferulic acid, which in the literature were found to be bioactive phenolic compounds that could help humans to ameliorate many diseases [37]. In tomatoes exposed to Zn and caffeine, the activation of a plant’s antioxidant system could be the result of the stimulation of the phenylpropanoid biosynthetic pathway, inducing the synthesis of the above-indicated phenolic acids [43]. It is important to note that some other molecules (the flavones apigenin and luteolin) were significantly reduced by Zn or caffeine. These two molecules are considered functional components in foods, and their relationship with health has been proven by numerous researchers [44]. In general, the increase in polyphenols under Zn + caffeine treatment could also be associated with tomatoes, with an increase in the total antioxidant capacity of the fruits after 56 days of treatment. For this reason, tomatoes treated with low levels of caffeine and Zn could be an excellent source of secondary metabolites that play beneficial roles in inhibiting reactive oxygen species by scavenging free radicals [37]. The ability of tomato plants to take up Zn or caffeine has important implications for human health risk assessment [28,45,46]. According to Kloke [47], Tf > 1 represents the capability to transfer the mineral element/organic compound in the aerial parts of the plants [48]. Under our experimental conditions, Zn and Zn + caffeine treatments showed Tf > 1 in the stem and leaves, indicating a clear translocation in the aerial part of the compost used. Therefore, it was necessary to evaluate the THQ-based risk assessment, as both compounds can be toxic to humans if taken in high concentrations. The THQ-based risk assessment method provides a more precise indication of risks [49,50]. THQ data for Zn ingestion indicated that there were no significant health risks associated with the intake of Zn- and caffeine-treated tomatoes. Moreover, our Zn THQ data are in line with a previous meta-analysis study on tomatoes irrigated with Zn-containing water [43]. Finally, the measured risk due to the ingestion of tomatoes irrigated with caffeine indicated that THQ values in all fruits were lower than 1, indicating no considerable health risks for human consumption [28]. 

## 5. Conclusions

In conclusion, under our experimental conditions, the quality of fruits was conserved, and for some antioxidant molecules, such as 4-coumaric, caffeic, and t-ferulic acid, the concentration was increased. The estimated THQ values demonstrated that the daily consumption of tomatoes irrigated with Zn- and caffeine-treated water used in this study did not pose a health risk. Further specific analyses must be performed, especially for the dose related to lycopene and beta-carotene, considering the impact of these compounds on the total antioxidant profile of tomatoes. We also acknowledge that the results of our short-term experiment are not comparable to field ones because plant responses may change over time due to complex plant–soil interactions but provide an indication of the positive effect of caffeine and Zn on tomato plants and could be taken under consideration for the future formulation of biostimulant products. 

## Figures and Tables

**Table 1:** Total soluble solids content (°Brix) and ethylene production (nl g^−1^ h^−1^) in *Solanum lycopersicum* ‘Panarea’ plants. One fruit per truss of the first, second, and third truss clusters was used for the analyses after 32, 48, and 56 days of treatment, respectively. Tap water (control), 0.136 mg L^−1^ Zn (Zn), 1 mg L^−1^ caffeine (trimethyl-^13^C) (caffeine), 1 mg L^−1^ caffeine (trimethyl-^13^C), and 0.136 mg L^−1^ Zn (Zn + caffeine). Data (*n* = 5) were analyzed using two-way ANOVA. Tukey’s post-hoc test at *p* ≤ 0.05 probability level was applied, and statistical data are reported in Supplementary Table S4.

		Treatments			
	Day of Treatment	Control	Zn	Caffeine	Zn + Caffeine
Total soluble solids content (°Brix)	32	8.9 ± 0.9	9.4 ± 1.4	8.8 ± 0.8	8.4 ± 1.2
	48	9.0 ± 1.0	10.0 ± 1.4	9.0 ± 0.8	9.0 ± 1.1
	56	10.4 ± 2.2	8.9 ± 0.8	9.0 ± 0.7	9.0 ± 1.4
Ethylene (nl g^−1^ h^−1^)	32	0.37 ± 0.3	0.40 ± 0.2	0.18 ± 0.1	0.52 ± 0.5
	48	0.16 ± 0.1	0.45 ± 0.5	0.13 ± 0.1	0.14 ± 0.1
	56	0.32 ± 0.3	0.48 ± 0.6	0.25 ± 0.2	0.44 ± 0.3

**Table 2:** Concentration (ng g^−1^ FW) of the selected polyphenols: protocatechuic acid (PCTA), naringenin (NRG), quercetin (QCT), chlorogenic acid (CGA), piceid (PCD), phloridzin (PDZ), kaempferol 7-G (QCT7G), kaempferol 3-G (QCT3G), kaempferol 3-O-rutinoside (KPF3R), rutin (RTN), quercetin 3,4 DG (QCTDG) analyzed in first fruit of first, second, and third truss of *Solanum lycopersicum* cv ‘Panarea’ treated with tap water (control), 0.136 mg L^−1^ Zn (Zn), 1 mg L^−1^ caffeine (trimethyl-^13^C) (caffeine), 1 mg L^−1^ caffeine (trimethyl-^13^C), and 0.136 mg L^−1^ Zn (Zn + caffeine). Data (*n* = 5) are expressed as the mean ± standard deviation.

			Treatments		
	Compound	Control	Zn	Caffeine	Zn + Caffeine
third Truss	PCTA	921 ± 220	722 ± 258	542 ± 294	863 ± 385
	NRG	747 ± 551	1935 ± 1312	1816 ± 2150	641 ± 212
	QCT	221 ± 139	259 ± 79	197 ± 61	204 ± 92
	CGA	125,798 ± 41,569	141,023 ± 42,128	131,831 ± 59,148	187,224 ± 62,235
	PCD	84 ± 25	111 ± 33	108 ± 26	98 ± 27
	PDZ	1584 ± 1146	2878 ± 1693	1939 ± 710	1878 ± 1201
	KPF7G	320 ± 29	374 ± 133	334 ± 209	303 ± 103
	KPF3G	65 ± 31	102 ± 44	94 ± 38	119 ± 64
	KPF3R	15,648 ± 5099	22,661 ± 4649	19,293 ± 10,572	20,822 ± 8325
	RTN	309,569 ± 97,652	375,194 ± 74,526	353,134 ± 72,683	365,085 ± 10,102
	QCTDG	916 ± 396	1263 ± 229	1304 ± 742	1283 ± 427
second Truss	PCTA	173 ± 117	833 ± 586	595 ± 153	709 ± 234
	NRG	76 ± 41	544 ± 818	299 ± 63	428 ± 421
	QCT	444 ± 245	204 ± 116	131 ± 27	94 ± 15
	CGA	8637 ± 5989	48,542 ± 23,516	47,921 ± 15,864	74,721 ± 45,677
	PCD	14 ± 10	67 ± 29	70 ± 26	80 ± 27
	PDZ	289 ± 238	719 ± 230	1304 ± 793	1466 ± 607
	KPF7G	81 ± 58	43 ± 23	41 ± 18	70 ± 56
	KPF3G	65 ± 14	85 ± 22	88 ± 15	88 ± 17
	KPF3R	120 ± 10	44 ± 5	142 ± 75	149 ± 93
	RTN	30,355 ± 20,331	153,394 ± 5505	161,355 ± 91,172	224,005 ± 94,321
	QCTDG	183 ± 143	799 ± 60	1309 ± 965	1127 ± 594
first Truss	PCTA	402 ± 170	522 ± 117	675 ± 152	1110 ± 422
	NRG	273 ± 117	405 ± 231	460 ± 112	757 ± 374
	QCT	863 ± 681	239 ± 81	166 ± 19	156 ± 50
	CGA	57,333 ± 29,331	92,037 ± 41,689	70,646 ± 22,055	123,978 ± 25,866
	PCD	43 ± 20	58 ± 18	58 ± 18	66 ± 25
	PDZ	457 ± 198	1049 ± 547	1123 ± 466	1206 ± 471
	KPF7G	124 ± 65	64 ± 13	74 ± 25	68 ± 41
	KPF3G	34 ± 14	36 ± 14	57 ± 19	140 ± 134
	KPF3R	3499 ± 1613	6151 ± 2594	6108 ± 2879	9657 ± 6951
	RTN	72,725 ± 32,123	151,346 ± 59,515	122,042 ± 32,231	222,966 ± 119,537
	QCTDG	367 ± 194	851 ± 756	1004 ± 513	2398 ± 3216

**Table 3:** Zn and caffeine average concentration expressed as fresh weight and target hazard quotient (THQ) values in first, second, and third truss clusters of *Solanum lycopersicum* ‘Panarea’ plants exposed to 0.136 mg L^−1^ Zn (Zn), 1 mg L^−1^ caffeine (trimethyl-^13^C) (caffeine) or 0.136 mg L^−1^ Zn and 1 mg L^−1^ caffeine (trimethyl-^13^C) (Zn + caffeine).

	Truss Clusters	Zn	Zn + Caffeine
Zn_average_ (mg kg^−1^ FW)	first	3.79	2.29
	second	3.71	1.99
	third	2.85	1.52
THQ of Zn	first	0.009	0.005
	second	0.008	0.004
	third	0.006	0.003
		**Caffeine**	**Zn + Caffeine**
Caffeine_average_ (ng g^−1^ FW)	first	0.072	0.059
	second	0.060	0.034
	third	0.181	0.460
THQ of caffeine	first	1.73 × 10^−5^	1.42 × 10^−5^
	second	1.44 × 10^−5^	8.16 × 10^−6^
	third	4.33 × 10^−5^	1.10 × 10^−4^

## Data Availability

Data is contained within the article and Supplementary Materials.

## Supplementary Materials

The following supporting information can be downloaded at: https://www.mdpi.com/article/10.3390/antiox13091100/s1, Figure S1: Schematic representation of experimental design and sampling; Table S1: Mass spectrometer parameters; Table S2: Statistic of stem length; Table S3: Statistic on fruit physiology; Table S4: Statistic of total soluble solids content and ethylene production; Table S5: Statistic of Zn concentration in roots, stem and, leaves; Table S6: Statistic of Zn concentration in fruits; Table S7: Data of mineral elements in fruits; Table S8: Statistic of mineral elements concentration in fruits; Table S9: Statistic of polyphenols data in fruits.
