# Supplementary material for: Effects of Caffeine, Zinc, and Their Combined Treatments on the Growth, Yield, Mineral Elements, and Polyphenols of Solanum lycopersicum L
Source: Antioxidants (Basel). 2024 Sep 11;13(9):1100. doi: 10.3390/antiox13091100 (PMC11428628; doi:10.3390/antiox13091100)
Supplement: Supplementary file 1 [file antioxidants-13-01100-s001.zip › antioxidants-3149735-supplementary.pdf]

Effects of caffeine, zinc, and their combined treatments on the growth, yield, mineral elements, and polyphenols of *Solanum lycopersicum* L.

Elena Vichi[1], Alessandra Francini[1*], Andrea Raffaelli[1,2], Luca Sebastiani[1]

[1] Institute of Crop Science (ICS), Scuola Superiore Sant'Anna, Piazza Martiri della Libertà 33, 56127 - Pisa, Italy; elena.vichi@santannapisa.it, a.francini@santannapisa.it, andrea1.raffaelli@santannapisa.it, luca.sebastiani@santannapisa.it

[2] Institute of Agricultural Biology and Biotechnology-National Research Council (IBBA-CNR), Via Moruzzi 1, 56124 - Pisa, Italy andrea1.raffaelli@santannapisa.it

* Correspondence: a.francini@santannapisa.it

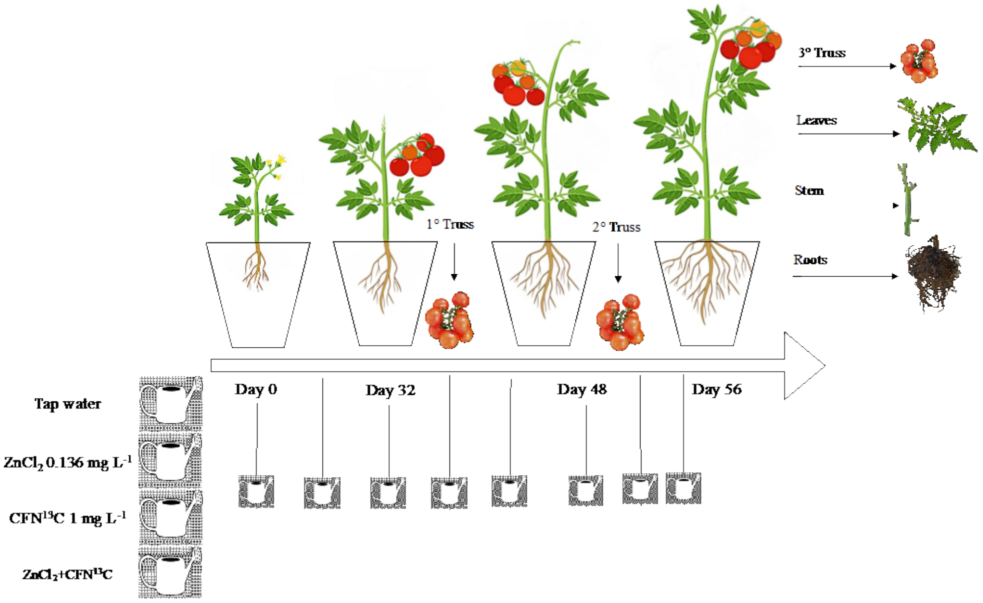

Figure S1. Schematic representation of experimental design and sampling (n = 7). Three trusses per plant were harvested at 32, 48 and 56 days after the start of the experiment at the red ripe stage. The sampled trusses underwent a total of 3, 6 and 8 treatments respectively and at the end of the experiment all part of the plant were sampled. One group was used as control and irrigated with tap water, the other three groups of plants were treated weekly with 100 ml of Zn (0.136 mg L^{-1}) or caffeine-(trimethyl-^{13}C) (1 mg L^{-1}) alone or a mix of both.

Citation: To be added by editorial staff during production.

Academic Editor: Firstname Lastname

Received: date
Revised: date
Accepted: date
Published: date

Copyright: © 2024 by the authors. Submitted for possible open access publication under the terms and conditions of the Creative Commons Attribution (CC BY) license (https://creativecommons.org/licenses/by/4.0/).

Table S1. A mass spectrometer operating in multiple reaction monitoring (MRM) mode was used to detect the investigated compound. The transitions were optimized for the compound-dependent parameters, including, two MRM transitions, declustering potential (DP), collision energy (CE), collision exit potential (CXP) and entrance potential (EP). Name, Acronym and Retention times are also reported.

Name	*Acronym*	*RT (min)*	*Q1*	*Q3*	*DP (V)*	*CE (eV)*	*CXP (V)*
Piceid	PCD	3.54	389.1	227	-125	-32	-11
Phloridzin	PDZ	4.01	535.1	272.9	-135	-23	-5
Luteolin	LTO	4.60	284.9	133	-130	-44.6	-17.4
Protocatechuic acid	PCTA	1.92	153	109	-86	-21	-7
4-Coumaric acid	4CL	3.35	163.0	119.0	-65	-18	-11
Caffeic acid	CFA	2.80	178.9	135.0	-86	-23	-11
t-Ferulic acid	TFRA	3.65	193.0	134.0	-62	-20	-8
Naringenin	NRG	5.13	270.9	150.9	-120	-25	-10
Apigenin	APG	5.02	268.9	117.0	-120	-49	-14
Catechin	CTC	2.69	289.0	244.9	-108	-22	-11
Quercetin	QCT	4.64	301.0	150.9	-113	-38	-8
Chlorogenic acid	CGA	2.56	353.0	151.0	-61	-24	-9
Kaempferol 7-O-glucoside	KPF7G	3.85	447.1	284.9	-158	-38	-5
Kaempferol 3-O-glucoside	KPF3G	3.69	447.1	284.1	-202	-39	-11
Rutin	RTN	3.29	609.2	299.9	-154	-48	-11
Quercetin 3,4-O-diglucoside	QCTDG	3.00	625.1	270.9	-178	-85	-12
Caffeine $^{13\text{-}}$C	CFN$^{13\text{-}}$C	2.13	198.1	140.1	-119	-28	-15

Table S2. The data for stem length was subjected to two-way ANOVA and P value are reported after Tukey's post hoc test at $P \leq 0.05$.

**ANOVA (p value)**		**Days**			
		**0**	**32**	**48**	**56**
**Stem length**	Zn	0.335	0.540	0.660	0.005
	Caffeine	0.852	0.330	0.638	0.588
	Caffeine x Zn	0.123	**0.023**	0.078	**0.035**

Table S3. The data about fruit yield (g/plant_FW), fruit number per plant, caliber (mm), DPPH(%) and one fruit DW (g) were subjected to two-way ANOVA and p value are reported in table after Tukey's post hoc test, $P \leq 0.05$.

ANOVA (p value)	Zn	Caffeine	Caffeine x Zn
Fruit yield (g/plant_FW)	**0.043**	0.347	0.284
Fruit number per plant	**0.001**	0.456	**0.009**
Caliber (mm)	0.810	0.570	0.906
DPPH (%)	0.934	**0.019**	0.573
One fruit DW (g)	0.272	**<0.001**	**0.001**

Table S4. Data (n = 5) for total soluble solids content (°Brix) and ethylene production (nl g^{-1} h^{-1}) were subjected to two-way ANOVA and P value are reported after Tukey's post hoc test, P ≤ 0.05.

**ANOVA (p value)**		**Days**		
		**32**	**48**	**56**
	Zn	0.951	0.560	0.467
**Total soluble solids content** (°Brix)	Caffeine	0.407	0.090	0.467
	Caffeine x Zn	0.363	0.580	0.935
	Zn	0.166	0.178	0.945
**Ethylene (nl g^{-1} h^{-1})**	Caffeine	0.769	0.082	0.632
	Caffeine x Zn	0.274	0.196	0.240

Table S5. Data about Zn concentration (mg kg^{-1} DW) in roots, stem and, leaves at day 56 were subjected to two-way ANOVA and P value are reported in table after Tukey's post hoc test, P ≤ 0.05.

**ANOVA (P value)**		**Roots**	**Stem**	**Leaves**
	Zn	**<0.001**	**0.0438**	**0.030**
**Zn (mg kg^{-1} DW)**	Caffeine	0.608	0.380	0.452
	Caffeine x Zn	**0.002**	0.838	0.588

Table S6. Data about Zn concentration (mg kg^{-1} DW) in fruits after 32, 48 and 56 days of treatments were subjected to two-way ANOVA and p value are reported in table after Tukey's post hoc test, P ≤ 0.05. The difference in the concentration between caffeine and mix treatment was analysed by t- test.

**ANOVA (p value)**		**Days**		
		**32**	**48**	**56**
	Zn	0.475	**0.001**	0.084
**Zn mg kg^{-1} DW**	Caffeine	0.847	**<0.001**	0.465
	Caffeine x Zn	0.988	**0.027**	**0.003**
**Caffeine ng g^{-1} FW**	*t*-test	0.182	0.139	**0.041**

Table S7. Concentrations of Cu, Mn, Fe, Na, Ca, Mg, K (mg kg^{-1} of dry weight), in the 3rd truss, 2nd truss and 1st truss of *Solanum lycopersicum* cv ‘Panarea’ after 56, 48 and 32 days of treatment with tap water (Control), 0.136 mg L^{-1} Zn (Zn), 1 mg L^{-1} Caffeine-(trimethyl-^{13}C) (Caffeine), 1 mg L^{-1} Caffeine-(trimethyl-^{13}C) and 0.136 mg L^{-1} Zn (Zn + Caffeine). Data (*n* = *7*) were expressed as means ± standard deviation.

		Elements						
	Treatments	Cu	Mn	Fe	Na	Ca	Mg	K
3th Truss	*Control*	*14* ± 4	*42* ± 9	*72* ± 16	*510* ± 121	*1160* ± 365	*1621* ± 374	*26243* ± 5650
	Zn	*14* ± 3	*39* ± 6	*62* ± 22	*486* ± 144	*1248* ± 264	*1904* ± 320	*32909* ± 4939
	Caffeine	*15* ± 1	*44* ± 5	*62* ± 14	*575* ± 94	*1401* ± 301	*2013* ± 385	*32951* ± 4627
	Zn + Caffeine	*13* ± 2	*38* ± 6	*48* ± 7	*527* ± 110	*1329* ± 515	*1776* ± 432	*31209* ± 5511
2nd Truss	*Control*	*14* ± 3	*51* ± 10	*60* ± 17	*493* ± 80	*1625* ± 534	*1766* ± 273	*27691* ± 2818
	Zn	*18* ± 8	*40* ± 8	*47* ± 15	*436* ± 100	*1322* ± 607	*1936* ± 512	*29503* ± 6743
	Caffeine	*11* ± 2	*33* ± 12	*42* ± 13	*408* ± 72	*1488* ± 293	*1800* ± 140	*31887* ± 4134
	Zn + Caffeine	*12* ± 3	*41* ± 9	*46* ± 12	*418* ± 77	*1641* ± 511	*1860* ± 342	*31024* ± 4707
1st Truss	*Control*	*11* ± 3	*43* ± 8	*59* ± 11	*369* ± 42	*2401* ± 643	*1915* ± 231	*31079* ± 2593
	Zn	*12* ± 2	*45* ± 12	*62* ± 10	*382* ± 96	*2136* ± 672	*1746* ± 265	*28167* ± 1840
	Caffeine	*12* ± 2	*42* ± 14	*54* ± 11	*373* ± 61	*2446* ± 559	*2048* ± 443	*32362* ± 3993
	Zn + Caffeine	*13* ± 4	*52* ± 13	*60* ± 14	*409* ± 71	*2436* ± 552	*1773* ± 320	*27585* ± 3874

Table S8. Mineral elements data (mg kg^{-1} DW) in fruits after 32, 48 and 56 days of treatments were subjected to two-way ANOVA and p value are reported in table after Tukey's post hoc test, $P \leq 0.05$

	**3rd Truss**						
**ANOVA (p value)**	**Cu**	**Mn**	**Fe**	**Na**	**Ca**	**Mg**	**K**
Zn	0.197	0.116	**0.006**	0.635	0.858	0.915	0.707
Caffeine	0.660	0.683	0.508	0.239	0.743	0.272	0.480
Zn x Caffeine	0.975	0.497	0.279	0.807	0.745	0.123	0.275
	**2nd Truss**						
**ANOVA (p value)**	**Cu**	**Mn**	**Fe**	**Na**	**Ca**	**Mg**	**K**
Zn	0.082	0.710	0.401	0.462	0.695	0.384	0.419
Caffeine	**0.008**	**0.034**	0.081	0.114	0.631	0.873	0.129
Zn x Caffeine	0.333	**0.024**	0.112	0.294	0.239	0.677	0.469
	**1st Truss**						
**ANOVA (p value)**	**Cu**	**Mn**	**Fe**	**Na**	**Ca**	**Mg**	**K**
Zn	0.368	0.165	0.333	0.369	0.556	0.083	**0.004**
Caffeine	0.396	0.578	0.419	0.563	0.460	0.520	0.775
Zn x Caffeine	0.909	0.407	0.631	0.667	0.584	0.666	0.448

Table S9. Polyphenols data (ng g^{-1} FW) in fruits after 32, 48 and 56 days of treatments were subjected to two-way ANOVA and p value are reported in table after Tukey's post hoc test, $P \leq 0.05$. Protocatechuic acid (PCTA), 4-coumaric acid (PCA), caffeic acid (CFA), t-ferulic acid (TFRA), naringenin (NRG), apigenin (APG), luteolin (LTO), quercetin (QCT), chlorogenic acid (CGA), piceid (PCD), phloridzin (PDZ), kaempferol *7*-G (QCT7G), kaempferol 3-G (QCT3G), kaempferol 3-O-rutinoside (KPF3R), rutin (RTN), quercetin *3,4* DG (QCTDG).

	**ANOVA (p value)**								
	3rd Truss			2nd Truss			1st Truss		
	**Zn**	**Caffeine**	**Zn x Caffeine**	**Zn**	**Caffeine**	**Zn x Caffeine**	**Zn**	**Caffeine**	**Zn x Caffeine**
**PCTA**	0.697	0.4310	0.0985	0.0184	0.3259	0.0822	0.023	0.0013	0.1739
**PCA**	0.4772	0.8607	0.5086	0.0378	0.0002	**0.0037**	0.0006	<0.0001	**0.0427**
**CFA**	0.866	0.683	0.686	0.0330	0.0007	**0.0251**	0.0003	<0.0001	0.2587
**TFRA**	0.3454	0.8304	0.9499	0.0244	0.0123	0.2325	0.0011	<0.0001	**0.0296**
**NRG**	0.952	0.915	0.061	0.1674	0.7987	0.4222	0.0578	0.0205	0.447
**APG**	0.1401	0.0969	0.3469	0.0202	0.0063	**0.0419**	0.0131	0.0041	**0.0376**
**LTO**	0.1316	0.0246	0.0891	0.0078	0.001	**0.0376**	0.0091	0.0021	0.0561
**QCT**	0.7993	0.3491	0.5429	0.0374	0.0031	0.1157	0.0558	0.0222	0.0633
**CGA**	0.1760	0.3100	0.4310	0.014	0.0156	0.5955	0.0054	0.1181	0.5063
**PCD**	0.7708	0.6263	0.1161	0.0096	0.0059	0.0697	0.2233	0.2179	0.675
**PDZ**	0.3404	0.5858	0.2542	0.2267	0.0018	0.5755	0.1063	0.0531	0.2153
**QCT7**	0.8774	0.7108	0.518	0.8059	0.7352	0.103	0.0951	0.235	0.1599
**QCT3G**	0.2404	0.3381	0.8064	0.2053	0.1316	0.2074	0.1826	0.0534	0.2022
**KPF3R**	0.2767	0.8393	0.3052	0.3422	0.0887	0.2526	0.107	0.1115	0.8081
**RTN**	0.313	0.668	0.313	0.0107	0.0064	0.3614	0.0117	0.0733	0.7283
**QCTDG**	0.9279	0.3805	0.3226	0.4641	0.0231	0.1863	0.5516	0.1642	0.228
